# TFAP2A enhances tumor stemness and promotes metastasis in pancreatic ductal adenocarcinoma

**DOI:** 10.1016/j.isci.2025.113060

**Published:** 2025-07-05

**Authors:** Jiaxin Luo, Zezhi Ding, Dongjie Chen, Yongsheng Jiang, Yizhi Cao, Minmin Shi, Xiaomei Tang, Jia Liu, Meilin Xue, Zehui Zhang, Kexian Li, Yu Bao, Fangfang Ma, Ting Wang, Lingxi Jiang

**Affiliations:** 1 Department of General Surgery, Pancreatic Disease Center, Ruijin Hospital, Shanghai Jiao Tong University School of Medicine, Shanghai, China; 2 Research Institute of Pancreatic Diseases, Shanghai Key Laboratory of Pancreatic Neoplasms Translational Research, Shanghai Jiao Tong University School of Medicine, Shanghai 200025, China; 3 Department of Pathology, Ruijin Hospital, Shanghai Jiao Tong University School of Medicine, Shanghai, China

**Keywords:** molecular biology, cell biology, cancer

## Abstract

Metastasis is the leading cause of death for nearly 90% of patients with cancer. In the digestive system, malignancies preferentially metastasize to the liver, which occurs in 76–80% of patients with pancreatic ductal adenocarcinoma (PDAC). Given the shared endoderm origin of embryonic pancreas and liver, this study investigated whether genes highly expressed in liver progenitors drive PDAC cells metastasize to the liver. Using an *in vitro* liver differentiation model, genes highly expressed in liver progenitors were identified. Among them, TFAP2A was highly expressed in PDAC and closely related to PDAC liver metastasis. Cancer associated fibroblasts (CAFs) upregulated TFAP2A expression by bone morphogenetic protein 4 (BMP4). Functional experiments demonstrated that TFAP2A overexpression promoted PDAC cell stemness and liver metastasis *in vitro* and *in vivo*. Mechanistically, TFAP2A could promote epithelial mesenchymal transition (EMT) and recruit macrophage by upregulating MYC, facilitating PDAC cell intravasation. Collectively, these findings unveil molecular mechanisms for PDAC liver metastasis and potential therapeutic targets.

## Highlights

• CAFs upregulate TFAP2A expression by BMP4 in PDAC
• TFAP2A promotes stemness and liver metastasis of PDAC cells
• TFAP2A recruits macrophages for the intravasation of PDAC cells by upregulating MYC
• TFAP2A knockdown enhances gemcitabine efficacy

## Graphical abstract

**Figure:**
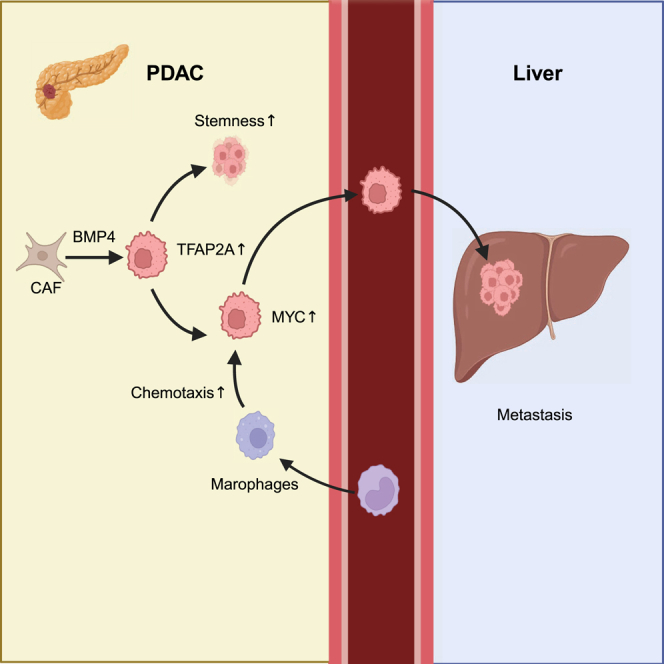

## Introduction

Pancreatic ductal adenocarcinoma (PDAC) is one of the most lethal malignancies with high incidence and poor prognosis in the world.^1^ In recent years, the incidence of PDAC has increased dramatically with mild survival improvement.^2^^,^^3^^,^^4^ The one-year survival rate of patients diagnosed with PDAC is less than 25%, and the 5-year survival rate is approximately 10%.^4^^,^^5^^,^^6^ About 76%–80% of patients with PDAC develop liver metastasis, which highly contributes to the poor prognosis of PDAC.^4^^,^^5^^,^^7^ Metastatic spread of tumors is a multistage process during which malignant cells disseminate from the primary tumor to distant organs.^8^^,^^9^ The “seed and soil” principle was elaborated by Stephen Paget to emphasize the crosstalk between primary tumor cells and remote metastatic organs.^10^ However, the understanding of how metastatic tumor cells (considered as “seeds”) adapt to and colonize remote metastatic organs (“soils”) is still limited.^11^

It has been reported that tissue-specific transcription factors or cell lineage genes could not only determine cell identity but also promote cancer metastasis to target organs via regulatory genomic elements, including promoters and enhancers.^12^^,^^13^^,^^14^ The pancreas and liver originate from the endoderm. Forming a foregut pocket, the definitive endoderm induces liver development via the release of FGFs and BMPs, while the pancreas is induced by different doses of FGFs from the foregut progenitors.^15^^,^^16^ The liver progenitors that highly express transcription factors, including FOXA1/2, HNF4A, SMAD2/3, function in a complex inter-regulatory network to control hepatocyte gene expression.^17^^,^^18^^,^^19^^,^^20^^,^^21^ Meanwhile, these factors have also been reported to contribute to the liver metastasis of cancer cells. FOXA2, a well-acknowledged liver lineage-determining transcription factor, is required for the expression of liver-specific genes and was upregulated in liver metastatic colorectal tumors.^22^ FOXA1 enhances PDAC cells’ invasiveness and metastatic potential *in vivo.*^23^ HNF4A suppresses tumor growth and drives PDAC cells toward an epithelial identity.^24^ SMAD2 enhances the metastatic ability of PDAC.^25^^,^^26^ These studies underscore the important roles of liver progenitor-specific transcription factors in liver metastasis of cancers.

In our previous study, we established an *in vitro* liver differentiation model with four developmental stages, including human embryonic stem cells (hESCs), definitive endoderm cells, liver progenitors, and pre-mature hepatocytes.^27^ Deep RNA sequencing of cells at these stages identified a group of genes that were specifically expressed in liver progenitors.^27^ Among these, PITX2 and RALYL have been demonstrated to enhance cell stemness and metastatic abilities in hepatocellular carcinoma,^28^^,^^29^ but the expression levels of PITX2 and RALYL were not associated with liver metastasis in PDAC (data not shown). Here, it was intriguing to find that TFAP2A, which exhibited expression patterns similar to other liver progenitor-specific genes in our liver differentiation model, was upregulated in liver metastatic tissues and clinically associated with liver metastasis of PDAC. Then, we confirmed the role of TFAP2A in promoting stemness and liver metastasis of PDAC through *in vivo* and *in vitro* functional experiments. Macrophage-derived granulin promotes the accumulation of myofibroblasts and PDAC growth at liver metastatic sites.^30^ Mechanistically, we found TFAP2A could activate the expression of MYC through direct promoter binding, thereby facilitating the recruitment of macrophages.

## Results

### The expression level of TFAP2A was associated with liver metastasis in pancreatic ductal adenocarcinoma

**Figure 1:**
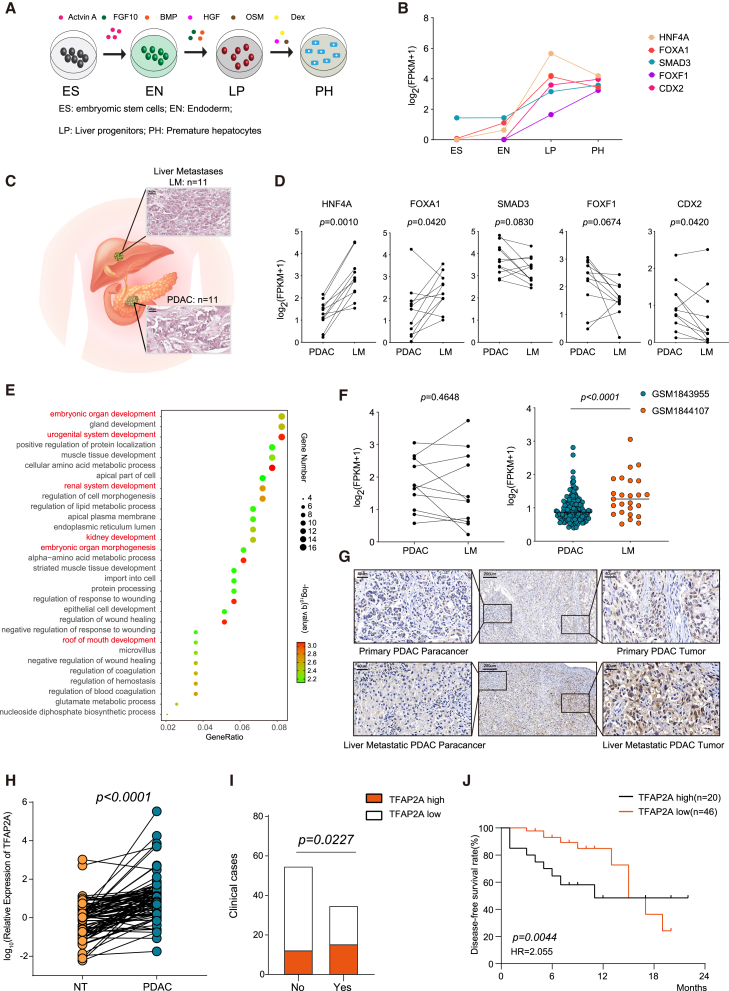
Identification of highly expressed genes in the liver progenitor of the *in vitro* liver differentiation model and clinical significance of TFAP2A expression (A) A schematic diagram of the previously established *in vitro* liver differentiation model with four stages. ES: embryonic stem cells; EN: Endoderm; LP: Liver progenitors; PH: Premature hepatocytes. (B) Expression levels of representative LP-specific highly expressing genes in the liver differentiation model. Also see Figure S1A. (C) Representative H&E staining images of PDAC and corresponding liver metastases (scale bar = 40 μm). (D) Expression levels of representative LP-specific highly expressing genes in PDAC and liver metastases samples from patients at Ruijin Hospital (*n* = 11). (E) GO enrichment analysis of genes with similar expression patterns to HNF4A and FOXA1 *in vitro* liver differentiation model. (F) Expression levels of TFAP2A in PDAC and liver metastases samples from patients at Ruijin hospital (*n* = 11), and in the GEO: GSM1843955 and GEO: GSM1844107 datasets. (G) Representative images of IHC staining of TFAP2A in primary PDAC and liver metastases. Scale bar = 200 μm or 40 μm. TFAP2A antibody dilution = 1:100. (H) Expression of TFAP2A in PDAC and adjacent normal tissue (NT) samples from 89 patients at Ruijin Hospital. (I) Stacked bar chart indicates the number of PDAC cases with or without liver metastasis in low or high expression of TFAP2A. No: without liver metastasis; Yes: with liver metastasis. Cutoff value = 23.43. (J) Kaplan–Meier disease-free survival curve of two groups of patients with PDAC at Ruijin Hospital. LM: liver metastasis. Statistical analyses were performed using Student’s t-tests.

We developed an *in vitro* liver differentiation model to induce endoderm (EN), liver progenitors (LPs), and premature hepatocytes (PHs) from embryonic stem (ES) cells^27^ (Figure 1A). Then, we performed RNA sequencing of cells derived from four stages in the *in vitro* model to profile the expression of protein coding genes in each stage (Table S1). The RNA sequencing results indicated that the expression of liver differentiation genes (*HNF4A, FOXA1, SMAD3, FOXF1*, *CDX2,* and so forth) gradually increased in LP and PH, while genes associated with pancreatic development (*PTF1A, NKX6-1, NEUROG3, INS, ARX,* and so forth) were absent or expressed at low levels in LP and PH (Figures 1B and S1A). Eleven pairs of PDAC tissues and their corresponding liver metastatic tissues were collected for hematoxylin-eosin (H&E) staining and RNA sequencing (Figure 1C). The RNA sequencing results demonstrated that the expression levels of genes associated with pancreatic development were significantly lower in liver metastatic tissues than in primary PDAC tissues (Figures S1A–S1C; Tables S2A and S2B). However, the key transcription factors of liver development (HNF4A and FOXA1) were upregulated in liver metastatic tissues compared with primary PDAC tissues (Figure 1D). Thereafter, a group of genes that had similar expression patterns to HNF4A and FOXA1 was filtered for further investigation (Table S3).

The enriched GO pathways of those genes mainly regulated organ development (Figure 1E; Table S4). The protein association network of filtered genes (https://string-db.org/)^31^ showed that TFAP2A had close interactions with other liver progenitor specific genes, including HNF4A and FOXA1 (Figure S1D). However, the roles of TFAP2A in liver metastasis in cancers have not been explored. Both RNA and protein levels of TFAP2A were significantly lower in primary PDAC tissues than their corresponding liver metastatic tissues (Figures 1F and 1G). Furthermore, RT-qPCR was performed on 89 samples of PDAC tissues together with their adjacent normal tissues to examine the expression levels of TFAP2A. The RT-qPCR results showed that TFAP2A was expressed at higher levels in PDAC tissues than in adjacent normal tissues (Figure 1H). As expected, the expression levels of TFAP2A in primary PDAC tissues were associated with liver metastasis, and patients with higher expression levels of TFAP2A had poorer disease free survival (Figures 1I and 1J; Table S5). The consistent conclusion could be drawn using the TCGA-PAAD dataset (Figure S1E). Taken together, higher expression of TFAP2A was associated with liver metastasis in PDAC.

### Cancer-associated fibroblasts upregulated the expression of TFAP2A through bone morphogenetic protein 4

**Figure 2:**
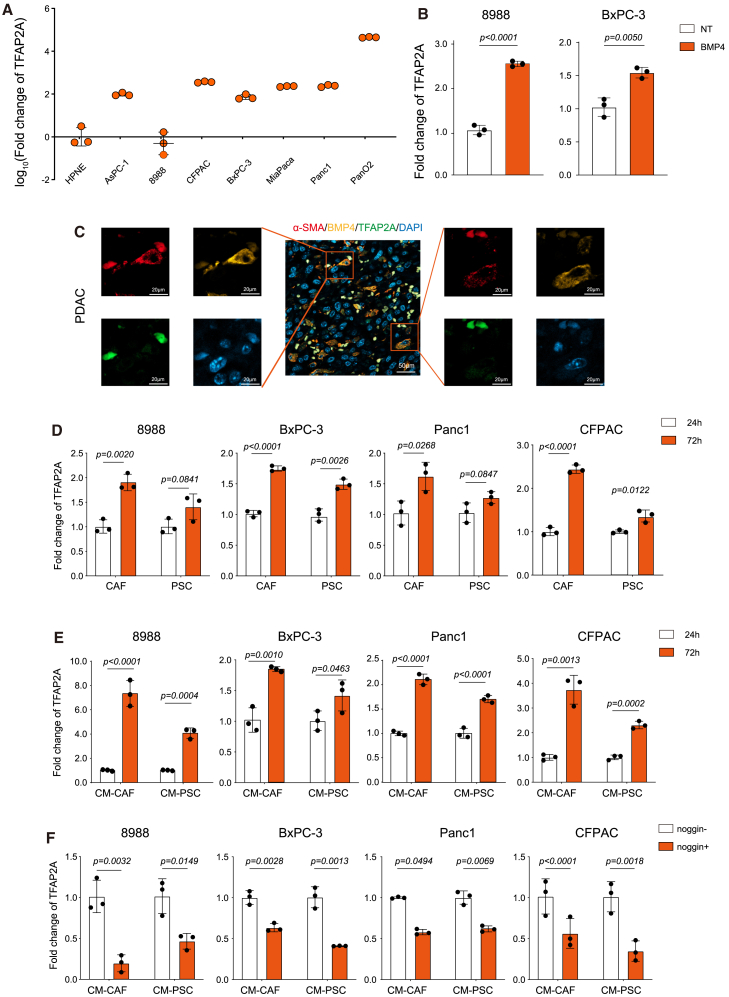
CAFs upregulate the expression of TFAP2A by BMP4 (A) Expression of *TFAP2A* in seven PDAC cell lines and the HPNE cell line. (B) Expression of *TFAP2A* in 8988 and BxPC-3 cells treated with BMP4 for 60 h. NT: no treatment (control). (C) Representative images of immunofluorescence staining of α-SMA (red), BMP4 (yellow), and TFAP2A (green) in PDAC. DAPI (blue) was used for nuclei counterstaining. Scale bar = 50 μm or 20 μm. Antibody dilution: α-SMA (1:100), BMP4 (1:100), TFAP2A (1:100). (D) Bar charts showing the fold change of *TFAP2A* in PDAC cell lines after directly co-culture with CAFs or PSCs for 24h or 72h. PDAC cells co-cultured for 24 h were used as controls. (E) Bar chart showing the fold change of *TFAP2A* in PDAC cell lines after being indirectly co-cultured with CAFs or PSCs for 24h or 72h. CM-CAF: conditional medium of CAFs; CM-PSC: conditional medium of PSCs. PDAC cells co-cultured for 24 h were used as controls. (F) Fold change of *TFAP2A* in PDAC cells treated with noggin or not. No noggin treatment was used as controls. All data are representative of at least three independent experiments. Error bars represent the mean values ±SD. Statistical analyses were performed using Student’s t-tests.

In the *in vitro* liver differentiation model, BMPs and FGFs were added to induce EN differentiation into LP, during which the expression of TFAP2A was activated (Figure 1A).^27^ During liver differentiation, TFAP2A showed a similar expression pattern to BMP4 (Figure S2A). Previous studies demonstrated that BMPs play crucial roles in all organ systems, especially in embryogenesis, where BMPs promote the formation of definitive endoderm in hESCs.^32^^,^^33^ BMP4 treatment increased the expression levels of TFAP2A during hESC differentiation.^34^ The expression levels of TFAP2A were checked in PDAC cell lines and HPNE cells. Among PDAC cell lines, 8988 and BxPC-3 showed relatively low expression of TFAP2A (Figure 2A). BMP4 treatment increased the expression of TFAP2A in these cell lines (Figure 2B). Single cell RNA sequencing (scRNA-seq) analysis of our previously published data (OEP003254)^35^ indicated that CAFs expressed the highest levels of BMP4 in the tumor microenvironment (Figure S2B). Additionally, immunofluorescence (IF) assays confrimed that BMP4 was highly expressed in CAFs adjacent to TFAP2A-positive cells (Figure 2C). To investigate whether CAFs could upregulate TFAP2A in tumor cells, PDAC cells were directly or indirectly co-cultured with CAFs. Both direct co-culture and conditional medium of CAFs increased TFAP2A expression in PDAC cells (Figures 2D and 2E). Moreover, the BMP4 antagonist noggin blocked CAF-induced TFAP2A upregulation (Figure 2F).

### TFAP2A contributed to the migration and stemness of pancreatic ductal adenocarcinoma cells

**Figure 3:**
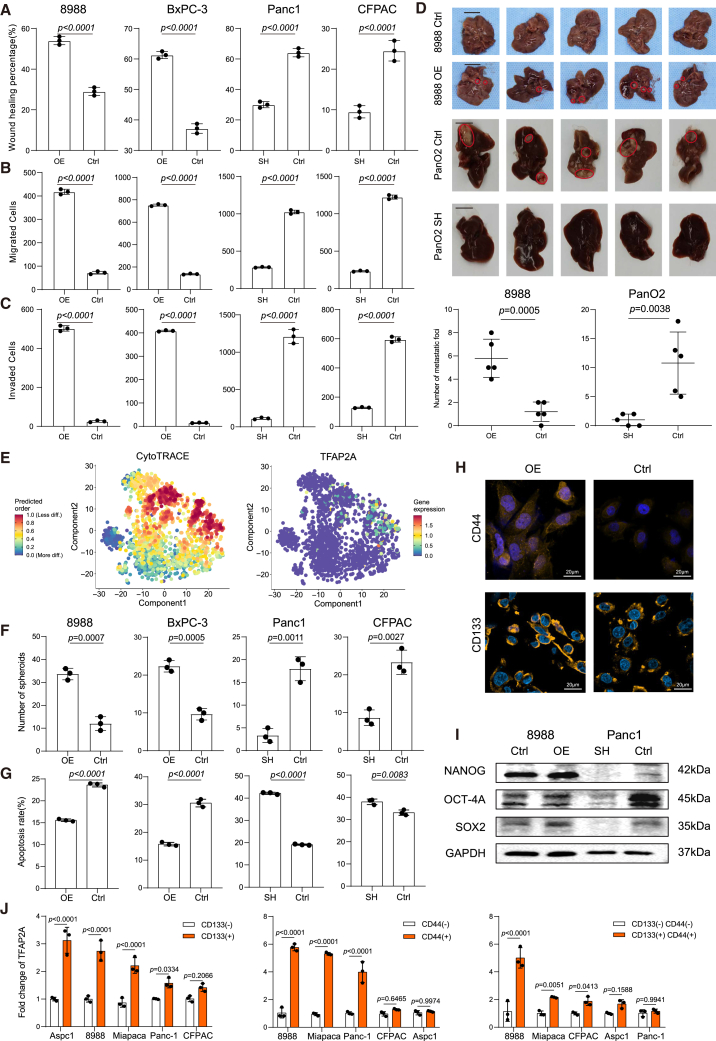
TFAP2A enhances migration and stemness capabilities of PDAC cells (A–C) The migration capability was measured via (A) wound healing assay, (B) transwell assay, and (C) invasion assay. The percentage of distance in wound healing assays, and the number of migrated or invaded cells are shown in a bar chart. The values represent the mean ± SD of three independent experiments (two-sided Student’s t test). Also see Figures S3A–S3C. (D) Liver metastases were excised at 12–16 weeks (8988 cells in BALB/c mice) or 6–8 weeks (Panc1 cells in C57BL/6 mice) after orthotopic pancreatic implantation (*n* = 5 per group). Representative images of excised livers are shown. Midlines show the number of metastatic foci developed by TFAP2A overexpression, knockdown, and control PDAC cells. OE: TFAP2A overexpression; SH: TFAP2A knockdown; Ctrl: control. Scale bar = 10mm. (E) CytoTRACE scores (left) and expression of TFAP2A (right) of ductal cells in PDAC using our previously published scRNA-seq data (OEP003254). (F) The number of spheroids formed by TFAP2A overexpression, knockdown, and control PDAC cells. OE: TFAP2A overexpression; SH: TFAP2A knockdown; Ctrl: control. Also see Figure S3F. (G) The apoptotic index of TFAP2A overexpression, knockdown, and control PDAC cells. OE: TFAP2A overexpression; SH: TFAP2A knockdown; Ctrl: control. Also see Figure S3H. (H) Representative images of the immunofluorescence staining of CD44 and CD133 in PDAC cells. DAPI (blue) was used for nuclei counterstaining. Scale bar = 20 μm. Antibody dilution: CD44 (1:100), CD133 (1:100). (I) The protein levels of NANOG, OCT-4A, and SOX2 in PDAC cells were determined via western blotting. GAPDH was used as an internal control. Antibody dilution = NANOG (1:2000), OCT-4A (1:1000), SOX2 (1:1000). (J) RT-qPCR results showing higher RNA levels of *TFAP2A* in CD133+, CD44^+^ and CD133+CD44^+^ PDAC cells than negative ones. Positive cells were sorted via a flow cytometry sorting system. Three independent experiments were performed. Error bars represent the mean values ±SD. Statistical analyses were performed using Student’s t-tests.

To explore the function of TFAP2A on tumor metastasis of PDAC cells, *in vitro* functional assays, including wound healing, transwell assays, and invasion assays, were performed. Considering the differential expression levels of TFAP2A among PDAC cell lines, 8988 and BxPC-3 were selected for TFAP2A overexpression system, while Panc1 and CFPAC were selected for knockdown assays (Figure S2C). TFAP2A overexpression promoted the migration ability of PDAC cells (Figures 3A–3C and S3A–S3C). *In vivo* liver metastasis models via pancreas orthotopic implantation were further established to investigate the potential role of TFAP2A in liver metastasis. Larger metastatic nodules and higher metastatic frequency were observed in cells with higher expression of TFAP2A (Figures 3D and S3D). A higher CytoTRACE score was associated with poorer differentiation.^36^ scRNA-seq analyses using our previously published data (OEP003254)^35^ indicated that ductal cells in PDAC with higher expression of TFAP2A had higher scores of CytoTRACE (Figure 3E), suggesting that TFAP2A could regulate cell stemness. Subsequently, stemness-related functional assays were performed, and the expression levels of stemness related genes were examined. TFAP2A increased the frequency and size of spheroids and organoids formed by PDAC cells (Figures 3F, S3E, and S3F). The IC50 values of gemcitabine were increased in PDAC cells with higher expression of TFAP2A compared to the controls (Figure S3G). Moreover, the apoptotic index of TFAP2A highly expressing cells decreased significantly (Figures 3G and S3H). Consistently, TFAP2A enhanced the protein levels of cancer stem cell markers and stemness-related genes, including CD44, CD133, NANOG, OCT-4A and SOX2 (Figures 3H, 3I, and S3I). Furthermore, the expression of TFAP2A was positively associated with CD44 (*R* = 0.46, *p* < 0.0001) and CD133 (*R* = 0.71, *p* < 0.0001) in PDAC clinical samples (Figure S3J). The ratio of CD133+CD44+ cells increased in cells expressing higher levels of TFAP2A (Figure S3K). Consistently, the expression of TFAP2A was increased in sorted CD133+, CD44+ and CD133+CD44+ cells from PDAC cell lines compared to negative cells (Figure 3J).

### TFAP2A promoted liver metastasis of pancreatic ductal adenocarcinoma by binding to the MYC promoter region and activating the epithelial mesenchymal transition process

**Figure 4:**
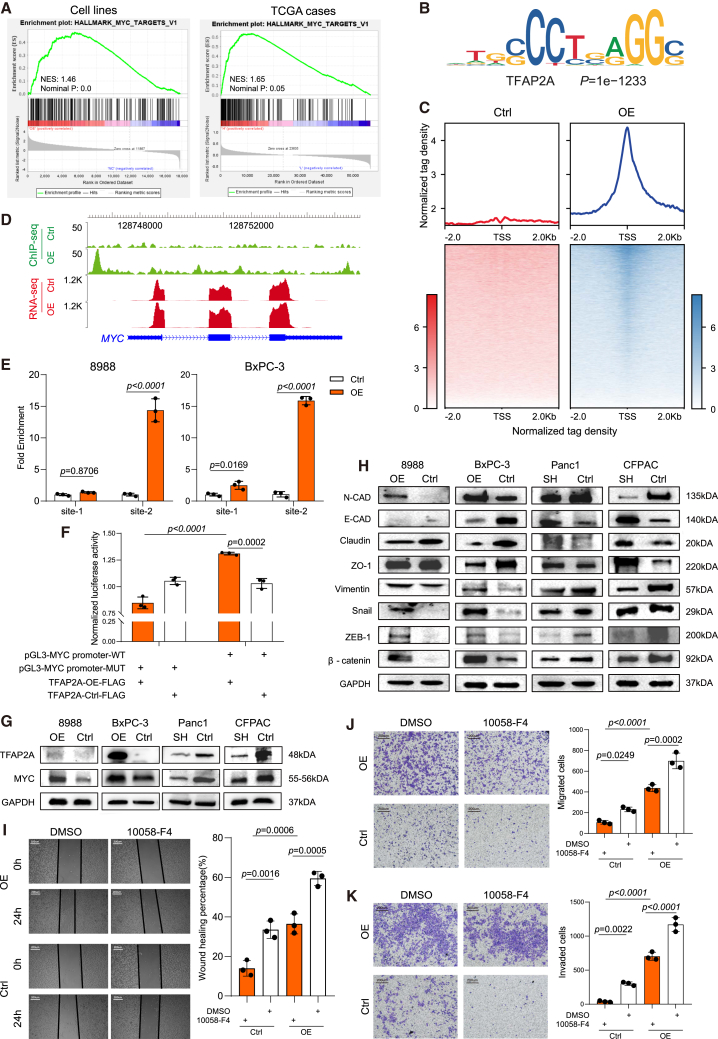
TFAP2A binds to the MYC promoter region and activates EMT (A) The gene set enrichment analysis (GSEA) of MYC comparing PDAC cell lines between TFAP2A overexpression and control groups or comparing two groups of patients divided by TFAP2A expression level from TCGA datasets. OE: TFAP2A overexpression group; NC: control group; H: TFAP2A high expression group; L: TFAP2A low expression group. (B) TFAP2A consensus motif identified in our FLAG-tagged TFAP2A ChIP-seq dataset. Statistical significance expressed as *p* value is shown. (C) Aggregation plots and heatmaps of ChIP-seq read densities in TFAP2A overexpression and control group centered by regions flanking 2 Kb of TSS. Results are normalized with the input of total sonicated chromatin. (D) ChIP-seq and RNA-seq signals near MYC in TFAP2A overexpression and control groups. ChIP-seq and RNA-seq profiles were visualized by Integrative Genomics Viewer (IGV). (E) ChIP-qPCR results for PDAC cell lines concerning the binding of TFAP2A to the MYC promoter. IgG was considered a control. (F) Quantitative analyses of dual-luciferase reporter assay in HEK293T. (G and H) The protein levels of (G) TFAP2A, MYC, and (H) EMT-related genes in PDAC cell lines were determined by western blotting. GAPDH was used as a loading control. Antibody dilution = 1:1000. (I–K) Representative images (left) and summarized bar chart (right) of wound healing assay (I), transwell assay (J), and invasion assay (K). Scale bar = 200 μm. Three independent experiments were performed. Error bars represent the mean values ±SD. Statistical analyses were performed using Student’s t-tests for two group’s comparisons and one-way ANOVA for multiple comparisons.

To further explore the mechanisms of TFAP2A in metastasis, RNA sequencing was performed on TFAP2A-overexpressing and control cells. GSEA analysis indicated that MYC signaling was upregulated in cells and PDAC clinical samples with higher TFAP2A expression (Figure 4A). ChIP-seq identified the TFAP2A-binding motif in TFAP2A-bound regions, and the majority of TFAP2A-binding sites were near the TSS (Figures 4B and 4C). ChIP-seq also indicated that the binding sites of TFAP2A were close to the MYC promoter region (Figure 4D). Potential binding sites of TFAP2A at the promoter region of MYC were predicted by JASPER (Table S6). ChIP-qPCR and dual-luciferase reporter assays further confirmed that TFAP2A could bind to specific site in the promoter region (Figures 4E and 4F). As is well known, MYC signaling plays an important role in tumorigenesis, tumor metastasis, and the EMT process.^37^^,^^38^^,^^39^ The protein levels of MYC and EMT markers were examined in PDAC cell lines (Figures 4G and 4H). To investigate whether the effects of TFAP2A on migration and invasion were mediated by MYC, the MYC inhibitor 10058-F4 was applied to TFAP2A-overexpressing cells. MYC inhibition significantly attenuated the migration and invasion capabilities of TFAP2A-overexpressing cells (Figures 4I–4K). Protein analysis indicated that the inhibition of MYC in TFAP2A-overexpressing cells attenuated the EMT process, while the reintroduction of MYC in TFAP2A-knockdown cells enhanced the EMT process (Figure S4A). Subsequent migration assays demonstrated that TFAP2A-knockdown cells exhibited improved migration capability after being transfected with MYC-overexpressing plasmids (Figures S4B and S4C).

### TFAP2A promoted liver metastasis of pancreatic ductal adenocarcinoma by indirectly recruiting macrophages to facilitate the intravasation of pancreatic ductal adenocarcinoma cells

**Figure 5:**
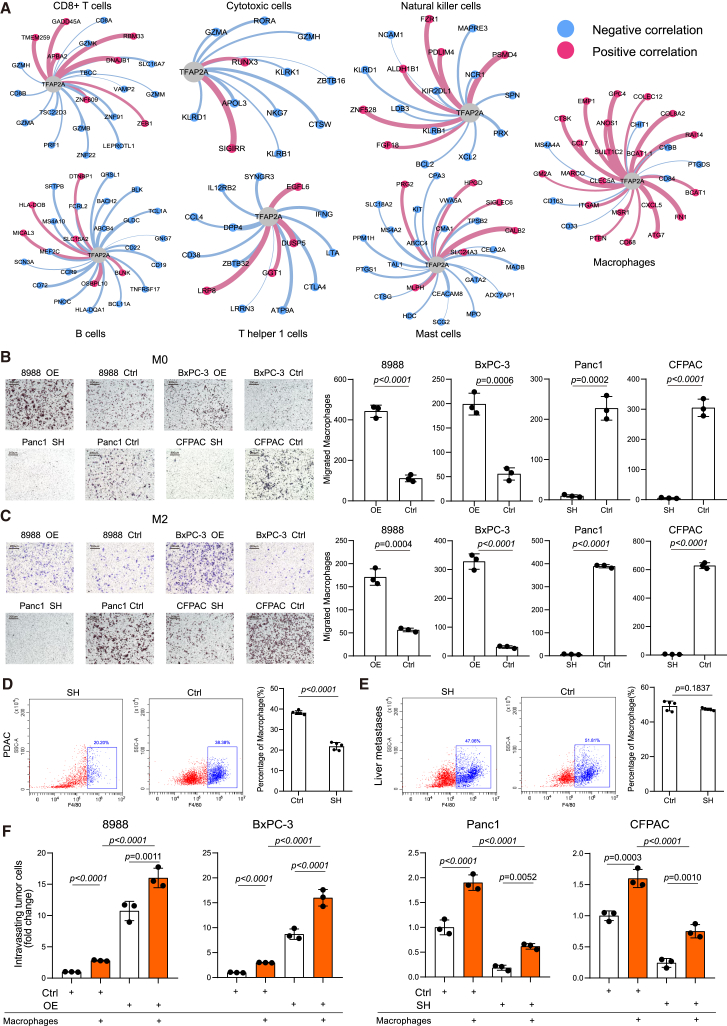
TFAP2A expression was associated with representative genes of macrophages and recruited TAMs to facilitate the intravasation of tumor cells (A) The Spearman’s correlation analysis of TFAP2A with representative genes of different immune cells in the tumor microenvironment of PDAC. The thickness of the line segment represents the magnitude of the correlation. (B) Representative images (left) of transwell assays and a summarized bar chart (right) of the number of migrated macrophages after co-culture with PDAC cells. Scale bar = 200μm. (C) Representative images (left) of transwell assays and a summarized bar chart (right) of the number of migrated M2-like macrophages. Scale bar = 200μm. (D and E) Flow cytometry was performed to examine the percentage of macrophages (F4/80+) in CD45^+^ immune cells in primary PDAC tissues (D) and the corresponding liver metastatic tissues (E) derived from Pan02 cells induced PDAC orthotopic implantation models in C57BL/6 (*n* = 5/group). The percentages of macrophages in CD45^+^ immune cells were summarized in bar charts (right). (F) The number of tumor cells migrating across the monolayer of HUVECs was summarized in a bar chart. iTEM assays were performed to investigate the intravasation capability of PDAC cells with or without macrophage incubation. Parental PDAC cells without macrophage incubation were used as a control. Three independent experiments were performed. Error bars represent the mean values ±SD. Statistical analyses were performed using Student’s t-tests for two group’s comparisons and one-way ANOVA for multiple comparisons.

As Maddipati et al. demonstrated that MYC promotes the intravasation of PDAC cells through TAM recruitment,^40^ we performed correlation analysis of TFAP2A expression and immune cells in the tumor microenvironment. As expected, macrophage marker genes positively correlated with TFAP2A expression (Figure 5A). Thus, we hypothesized that TFAP2A could recruit macrophages by upregulating MYC. Macrophage migration assays demonstrated that PDAC cells with higher expression of TFAP2A could promote macrophage and TAM (M2-like subtype) migrate through transwell chambers (Figures 5B and 5C). Moreover, Pan02 induced orthotopic tumor tissues from C57BL/6 mice were digested into single cells and analyzed by flow cytometry. We found that the percentages of macrophages decreased in primary tumors from TFAP2A-knockdown cells compared to controls, though no significant difference was observed in liver metastases (Figures 5D and 5E). Then, we performed an *in vitro* transendothelial migration (iTEM) assay^40^ to investigate whether macrophages could promote the intravasation of PDAC cells. The results indicated that both macrophages and TFAP2A expression enhanced the intravasation of PDAC cells, with optimal intravasation occurring when both factors were present (Figure 5F). Furthermore, to identify potential macrophage recruitment factors, ELISA assays for cytokines/chemokines identified by Maddipati et al. were performed.^40^ The results demonstrated that three factors (CCL3, CXCL3, and MIF) were enriched in the PDAC cell line with upregulated TFAP2A expression (Figure S5A).

To evaluate the clinical potential of TFAP2A inhibition, we combined TFAP2A knockdown with gemcitabine treatment *in vivo*. The results showed that the TFAP2A-knockdown group exhibited lower tumor burden and reduced liver metastasis incidence when combined with gemcitabine, compared to controls (Figures S5B–S5D).

## Discussion

Initially, we performed RNA sequencing of cells derived from four stages of the previously established *in vitro* liver differentiation model,^27^ and identified a group of genes with specific high expression in the LP and PH stages. Among them, TFAP2A was found to be associated with liver metastasis in PDAC. TFAP2A, as a transcription factor, has been reported to play important roles in the development and progression of various tumors.^41^^,^^42^^,^^43^ However, the roles of TFAP2A in PDAC pathogenesis and progression remained unclear. In our study, we characterized the functional role of TFAP2A in stemness and metastasis of PDAC, revealing that TFAP2A could promote migration, metastasis, and intravasation of PDAC cells by upregulating MYC and recruiting macrophages in primary tumors.

CAFs, one of the most prevalent cell types in the tumor microenvironment of PDAC, could upregulate the expression of TFAP2A through BMP4. Given the crucial role of BMP4 in embryogenesis,^32^^,^^33^ it was included in the established *in vitro* liver differentiation model to induce EN to LP differentiation. Unsurprisingly, the expression levels of TFAP2A were significantly increased in LP compared to EN. In the context of PDAC, high-throughput analyses, including RNA sequencing and ChIP sequencing, showed MYC upregulation when TFAP2A bound to its promoter region. MYC signaling is well acknowledged to be involved in tumorigenesis, tumor metastasis, and the EMT process.^37^^,^^38^^,^^39^ Maddipati et al. proved that MYC promotes the intravasation of PDAC cells through TAM recruitment.^40^ Consistent with the previous findings, we confirmed that TFAP2A could recruit macrophages to facilitate the intravasation of PDAC cells by upregulating MYC. Our study also demonstrated that TFAP2A knockdown could enhance gemcitabine efficacy, suggesting potential therapeutic benefits of combining TFAP2A inhibition with standard chemotherapy in future clinical practice.

In conclusion, upregulated by BMP4 derived from CAFs, TFAP2A could promote the stemness, migration, and metastasis of PDAC cells, recruit macrophages to facilitate the intravasation of PDAC cells, and finally enhance liver metastasis in PDAC via upregulating MYC. These results provide essential clues to further delineate the underlying mechanisms of liver metastasis in PDAC and shed light on exploring novel therapeutic targets for preventing liver metastasis in cancers.

### Limitations of the study

There is still a need for an in-depth investigation of the underlying mechanism of TFAP2A in PDAC liver metastasis. First, the molecular mechanism by which BMP4 regulates TFAP2A expression warrants subsequent intensive study. Second, since TFAP2A is specifically highly expressed in LP, whether TFAP2A can promote the differentiation of PDAC cells in the liver metastatic sites is also worthy of deeper consideration. Finally, it is worthwhile to delve into the interaction between high TFAP2A expressing PDAC cells and other cell types in the microenvironment of the liver metastasis.

## Resource availability

### Lead contact

Requests for further information and resources should be directed to and will be fulfilled by the lead contact, Lingxi Jiang (jlx12120@rjh.com.cn).

### Materials availability

This study did not generate new unique reagents.

### Data and code availability

• RNA-seq data have been deposited at NCBI as NCBI: PRJNA1262147 and are publicly available as of the date of publication.
• This article does not report original code.
• Any additional information required to reanalyze the data reported in this article is available from the lead contact upon request.

## Acknowledgments

Funding Statement This study is supported by the 10.13039/501100001809National Natural Science Foundation of China, China (82173219 and 82303266).

## Author contributions

L.J. and J.L. initiated and designed the experiments; J.L, Z.D., Y.C., M.S., X.T, and J.L. performed the experiments; D.C, J.L., and Z.D. analyzed the data and interpreted the data; Y.J. provided the PDAC clinical samples and the relevant clinical information; T.W, Z.Z, K.L., and F.M. provided valuable comments; J.L. drafted the article; Z.D., Y.B., and L.J. revised the article. T.W. and L.J. supervised the study. All authors read and approved the final article.

## Declaration of interests

The authors declare no competing interests.

## STAR★Methods

### Key resources table

**Table:** 

REAGENT or RESOURCE	SOURCE	IDENTIFIER
**Antibodies**		

Anti-TFAP2A	Abcam	Cat#ab108311; RRID:AB_10861200
Anti-alpha smooth muscle Actin	Abcam	Cat#ab5694; RRID:AB_2223021
Anti-MYC	Abcam	Cat#ab32072; RRID:AB_731658
Anti-BMP4	Invitrogen	Cat#PA5-27288; RRID:AB_2544764
Anti-TFAP2A	Invitrogen	Cat#MA1-872; RRID:AB_2199412
Anti-CD44	Proteintech	Cat#60224-1-Ig; RRID:AB_11042767
Anti-CD133	Proteintech	Cat#18470-1-AP; RRID:AB_2172859
Anti-CD90	Proteintech	Cat#66766-1-Ig; RRID:AB_2882112
Anti-*c*-Kit	Cell Signaling Technology	Cat#3074; RRID:AB_1147633
Anti-N-Cadherin	Cell Signaling Technology	Cat#13116; RRID:AB_2687616
Anti-E-Cadherin	Cell Signaling Technology	Cat#3195; RRID:AB_2291471
Anti-Claudin-1	Cell Signaling Technology	Cat#13255; RRID:AB_2798163
Anti-ZO-1	Cell Signaling Technology	Cat#8193; RRID:AB_10898025
Anti-Vimentin	Cell Signaling Technology	Cat#5741; RRID:AB_10695459
Anti-Snail	Cell Signaling Technology	Cat#3879; RRID:AB_2255011
Anti-ZEB1	Cell Signaling Technology	Cat#3396; RRID:AB_1904164
Anti-β-Catenin	Cell Signaling Technology	Cat#8480; RRID:AB_11127855

**Chemicals, peptides, and recombinant proteins**		

Recombinant human BMP4 protein	Abcam	Cat#ab226417
Noggin protein	MedChemExpress	Cat#HY-P70558
10058-F4	MedChemExpress	Cat#HY-12702
CSF1	MedChemExpress	Cat#HY-P7050A

**Critical commercial assays**		

EpiTect ChIP OneDay Kit	Qiagen	Cat#334471
Dual Luciferase Reporter Gene Assay Kit	Yeasen	Cat#11402ES
SteadyPure Quick RNA Extraction Kit	Accurate Biotechnology	Cat#AG21017
Evo M-MLV RT Kit with gDNA clean for qPCR	Accurate Biotechnology	Cat#AG11705
SYBR® Green Premix Pro Taq HS qPCR Kit	Accurate Biotechnology	Cat#AG11701
PE Annexin V Apoptosis Detection Kit I	BD Biosciences	Cat#559763

**Deposited data**		

RNA-seq data	NCBI	PRJNA1262147

**Experimental models: Cell lines**		

8988	ATCC	N/A
Panc1	ATCC	N/A
BxPC-3	ATCC	N/A
CFPAC	ATCC	N/A
Pan02	ATCC	N/A
HUVEC	ATCC	N/A

**Experimental models: Organisms/strains**		

Mouse: Nude BALB/c	Phenotek	N/A
Mouse: C57BL/6	Phenotek	N/A

**Oligonucleotides**		

Primer: TFAP2A-qF: CAAGTACGAGGACTGCGAGG	This paper	N/A
Primer: TFAP2A-qR: GCTCGTGTAGGGAGATTGACC	This paper	N/A
Primer: GAPDH-F: GGAGCGAGATCCCTCCAAAAT	This paper	N/A
Primer: GAPDH-R: GGCTGTTGTCATACTTCTCATGG	This paper	N/A
Primer: Binding Site 1-F: CTACTGGGCTGGGGTATCAGG	This paper	N/A
Primer: Binding Site 1-R: CTGGAGTGCAGTGGCACAATC	This paper	N/A
Primer: Binding Site 2-F: AGCAAAATCCAGCATAGCGATTGG	This paper	N/A
Primer: Binding Site 2-R: CTTCCAAATCCGATGCACTGCAC	This paper	N/A
Primers for MYC promoter, see Table S7	This paper	N/A

**Recombinant DNA**		

Plasmid: TFAP2A knockdown	This paper	N/A
Plasmid: TFAP2A overexpression	This paper	N/A
Plasmid: pGL3-MYC promoter-WT	This paper	N/A
Plasmid: pGL3-MYC promoter-MUT	This paper	N/A

**Software and algorithms**		

GraphPad Prism v8.0.1	GraphPad Software	https://www.graphpad.com/
SPSS v20.0	IBM	https://www.ibm.com/cn-zh/spss
Gephi	Gephi Software	https://gephi.org/
ImageJ	NIH	https://imagej.nih.gov/ij/

### Experimental model and study participant details

#### Patient samples

We collected PDAC tissues with adjacent normal tissues and corresponding liver metastatic tissues from patients at the Ruijin Hospital affiliated with Shanghai Jiao Tong University School of Medicine, Shanghai, China between December 2018 and May 2021. All enrolled patients met the following criteria: (1) pathologically diagnosed with PDAC; (2) definitively diagnosed with liver metastasis through peritoneoscopy; (3) had a complete clinicopathological and follow-up information; and (4) had no other signs of distant metastasis except liver metastasis. A total of 89 pairs of PDAC with adjacent normal tissues and 11 pairs of PDAC with corresponding liver metastatic tissues were obtained from surgical specimens for HE, IHC and RT-qPCR.

#### Ethics statement

All institutional and national guidelines for the care and use of laboratory animals were followed (IACUC No. RJ2023010). All procedures followed were in accordance with the ethical standards of the responsible committee on human experimentation (institutional and national) and with the Helsinki Declaration of 1964 and later versions. This study was approved by the Ruijin Hospital Ethics Committee (reference number: 2013–70), and all participants involved in this study provided their written informed consent.

#### Cell culture

Human-derived PDAC cell lines 8988 (Patu8988t), Panc1, CFPAC, BxPC-3 and murine-derived PDAC cell line Pan02 were purchased from the American Type Culture Collection (ATCC, Virginia, USA). CAFs were isolated from human tumor tissues as previously described.^30^ All cell lines were tested for mycoplasma contamination. STR DNA profiling was performed for cell line authentication. Cells were cultivated in DMEM, IMDM or RPMI 1640 medium (Meilunbio, Liaoning, China) supplemented with 10% fetal bovine serum (FBS) (Gibco, California, USA) and 1% penicillin-streptomycin in a standard 37°C incubator with 5% CO_2_.

#### *In vivo* liver metastasis model

5-week-old male nude BALB/c mice and C57BL/6 mice were purchased from Phenotek (Shanghai, China) and fed in specified pathogen free (SPF) environment. Patu8988t with TFAP2A overexpression or Pan02 with TFAP2A knockdown and the controls were orthotopically implanted into pancreas of 5-week-old male nude BALB/c mice (10^7^ cells/mouse, 5 mice/group) or 5-week-old male C57BL/6 mice (10^7^ cells/mouse, 5 mice/group), respectively. Mice were randomly assigned to experimental and control groups. After mice were sacrificed, primary PDAC tissues and liver metastatic nodules were dissected and photographed. All animal experiments were approved by the Animal Care and Use Committee of Shanghai Jiao Tong University School of Medicine.

#### Data availability statement

All data used and analyzed in the paper are available within the manuscript and supplementary materials. Additional data related to this paper may be requested from the corresponding authors.

### Method details

#### Quantitative real-time PCR (RT-qPCR)

Reverse transcription was performed using the Evo M-MLV RT Kit with gDNA clean for qPCR (Accurate Biotechnology, Hunan, China). The RT-qPCR was performed using SYBR Green Premix Pro Taq HS qPCR Kit (Accurate Biotechnology, Hunan, China) in a real-time fluorescent quantitative PCR machine (Analytik Jena, Jena, Germany). GAPDH was used as the reference control. The primer sequences used in this study are shown in key resources table.

#### Immunohistochemistry (IHC) staining

The tumor tissues collected from patients at the Ruijin Hospital and *in vivo* liver metastasis model were formalin-fixed, and then paraffin-embedded. The paraffin-embedded sections were subjected to IHC staining according to the standard procedures. The sections were incubated with TFAP2A antibody (Abcam, ab108311) followed by HRP conjugated Goat Anti-Rabbit IgG (Servicebio, Guangzhou, China) and diaminobenzidine (DAB, Servicebio, Guangzhou, China). Finally, the slides were stained with hematoxylin (Solarbio, Beijing, China), sealed with neutral resins and photographed under microscope.

#### Co-culture assays

Cell co-culture assays include direct and indirect cell co-culture assays.

Direct co-cultures were established as follows: PDAC cell lines transfected with plasmid containing a puromycin resistance gene were co-cultured with pancreatic stellate cells (PSCs) and cancer associated fibroblasts (CAFs) using 6-well plates (Corning, Shanghai, China) for 24 to 72 h. PDAC cell lines treated with puromycin for 24 h or 72 h were chosen to extract RNAs. Indirect co-cultures were performed using conditional medium (CM) of CAFs or PSCs. PSC and CAF cells were cultured to 90% confluence and then cultured in serum-free medium for 24h to harvest CM. PDAC cells were incubated with CM-CAF or CM-PSC for 24 to 72 hours followed by RNA extraction. RT-qPCR was used to detect the expression of TFAP2A at mRNA level in PDAC cells.

#### Cell transfection and treatment

Overexpression and shRNA plasmids of TFAP2A and their negative controls were synthesized by BioeGene Co., Ltd. (Shanghai, China). Transfections were performed using the Hilymax (DOJINDO, Kumamoto, Japan). Transfected cells were harvested and validated for further experiments. PDAC cells were treated with different concentrations of gemcitabine to determine their IC50 values. 10058-F4 (MedChemExpress, HY-12702) was used as MYC inhibitor to block MYC activity by targeting MYC-Max interaction.

#### Cell migration assay

The migration capacity was evaluated by wound healing, transwell and invasion assays.

For the wound healing assay, transfected cells were cultured to 90% confluence, then wounds were scratched using tips. Images of the same area in the wound were captured at 0 and 24 h under a microscope after scratch. Transwell assays were performed using 24-well transwell chambers (Corning, Shanghai, China). 700 μL culture medium supplemented with 20% FBS was placed in the lower chamber, while 2–10×10^4^ cells suspended in 200 μL serum-free medium were added into the upper chamber. 24 h after seeding, the cells migrating to the lower chamber were harvested, fixed, and stained with 1% crystal violet solution for 20 min at room temperature. Invasion assays were performed using 24-well transwell chambers, in which the upper layer was coated with matrigel (Corning, Shanghai, China). The remaining steps are the same with transwell assays.

#### Spheroid formation

PDAC cells were resuspended in the serum-free medium and seeded in Ultra-Low Attachment 24-Well Plates (Corning, Shanghai, China) (2 000–4 000 cells/well) in 300 μL of 3D Tumorsphere Medium XF (PromoCell, Heidelberg, Germany). Medium was changed every 2 days. After 5–6 days, spheroids were enzymatically and mechanically dissociated and collected by centrifugation at 500× g for 5 min. The resulting single cells were resuspended and seeded in new Ultra-Low Attachment 24-Well Plates (500–1 000 cells/well). After 7 days, images of the formed spheroids (diameter ≥ 50 μm) were observed and counted under Axio V ertA1 inverted microscope (Carl Zeiss, Oberkochen, Germany). The efficiency of spheroid formation was determined on the basis of the size and number of spheroids.

#### Flow cytometry analysis

Flow cytometry was used to detect cell apoptosis after gemcitabine treatment, surface proteins of PDAC cells and macrophages in tumor tissues derived from mice.

For cell apoptosis, transfection and control cells were treated with gemcitabine (IC50 concentration) for 48–72 h. Cells adhered to the bottom surface and floated in the cell culture supernatant were collected for cell apoptosis assays. The PE Annexin V Apoptosis Detection Kit I (BD Biosciences, New Jersey, USA) was performed according to the manufacturer’s instructions. Flow cytometry analysis was performed on a CytoFLEX5 flow cytometer (Beckman, California, USA). For surface proteins detection, APC anti-human CD44 Antibody (Bio Legend, California, USA) and PE anti-human CD133 Antibody (Bio Legend, California, USA) were used to examine the expression of CD44 and CD133 of PDAC cells. For macrophages detection, tumor tissues derived from C57BL/6 mice models were cut into small pieces, digested into single cells and resuspended in PBS. Then the CD45^+^ cells were isolated using immunomagnetic columns (Miltenyi Biotec, Bergisch Gladbach, Germany). The percentage of macrophages (F4/80+, Bio Legend, California, USA) was calculated in CD45^+^ immune cells.

#### Immunofluorescence (IF)

Tissue sections were dewaxed with xylene and ethanol, permeabilized in phosphate-buffered saline (PBS) and blocked with 5% bovine serum albumin (BSA). The sections were then incubated with primary antibodies targeting α-SMA (Abcam, ab5694), BMP4 (Invitrogen, PA5-27288), TFAP2A (Invitrogen, MA1-872), CD44 (Proteintech, 60224-1-Ig), CD133 (Proteintech, 18470-1-AP), CD90 (Proteintech, 66766-1-Ig) or c-kit (Cell Signaling Technology, 3074) overnight at 4°C. HRP-labeled goat anti-mouse/rabbit IgG secondary antibodies were applied for 1 h at room temperature. Sections were mounted with Vectashield mounting medium with DAPI. Images were acquired via confocal microscope (Carl Zeiss, Oberkochen, Germany).

#### RNA sequencing (RNA-seq)

RNA-seq was performed by Majorbio (Shanghai, China). In brief, the workflow consists of RNA enrichment, cDNA library preparation, size selection, and PCR amplification. The total RNA in TFAP2A overexpression PDAC cells and control group were extracted using SteadyPure Quick RNA Extraction Kit (Accurate Biology, Hunan, China). The size selection of cDNA used AMPure XP Beads (Beckman, California, USA). Sequencing was performed using an Illumina NovaSeq X Plus (Illumina, California, USA). The results of RNA-seq were analyzed on Majorbio analysis platform.

#### Chromatin immunoprecipitation (ChIP)

ChIP was performed according to the manufacturer’s instructions (Qiagen, Dusseldorf, Germany). Briefly, cells transfected with TFAP2A-FLAG plasmids and control ones were crosslinked using formaldehyde for 10 min at 37°C and added glycine solution to stop the crosslink reaction. Lysis buffer was applied to liberate cellular components. DNA fragmentation was obtained mechanically by sonication and immunoprecipitated using anti-FLAG antibody (Cell Signaling Technology, Boston, USA). Then, the target DNA-protein complex was isolated. Proteinase K was used to remove proteins from the complex and purified DNA was harvested. The purified DNA was further applied for RT-qPCR and sequencing.

#### Dual-luciferase reporter assay

The binding sites of TFAP2A and the promoter region of MYC were predicted using JASPER (http://jaspar.genereg.net/). Among the predicted binding sites, the top two were selected for further validation. The pGL3 luciferase reporter vectors were used to construct luciferase reporter plasmids (BioeGene Co.,Ltd, Shanghai, China). HEK293T cells were transfected with pGL3-MYC promoter-WT or pGL3-MYC promoter-MUT plasmids (sequences are shown in Table S7) and co-transfected with TFAP2A overexpression plasmid or negative control. Dual Luciferase Reporter Gene Assay Kit (Yeasen, Shanghai, China) was used to measure luciferase activity, and Renilla luciferase was used for normalization.

#### Western blotting

RIPA lysis buffer mixed with protease and phosphatases inhibitors cocktail was used to extract proteins from cells. Protein samples were separated using 10% SDS-PAGE and transferred to PVDF membranes. Primary and secondary antibodies were used as follows: TFAP2A (Abcam, ab108311), MYC (Abcam, ab32072), EMT Antibody Sampler Kit (Cell Signaling Technology, 9782T), GAPDH (Proteintech, 60004-1-Ig), and HRP-linked anti-rabbit IgG Antibody (Cell Signaling Technology, 7074), HRP-linked anti-mouse IgG Antibody (Cell Signaling Technology, 7076).

#### Macrophage transwell migration assay

Macrophage transwell migration assay was performed using 12-well transwell chambers (Corning, Shanghai, China) coated with matrigel (Corning, Shanghai, China). 2–5×10^5^ TFAP2A-overexpressing or knockdown cells were plated to the bottom of the chamber, while 1×10^5^ macrophages were added into the upper chamber. After 24–48 h, the macrophages migrated to the lower chamber were harvested, fixed, and stained with a 1% crystal violet solution for 20 min at room temperature. The remaining steps were performed as previously described.^40^

#### *In vitro* transendothelial migration assay (iTEM)

*In vitro* Transendothelial Migration assay used 12-well transwell chambers covered with matrigel. Human Umbilical Vein Endothelial cells (HUVEC) (purchased from ATCC) (1×10^4^ cells/well) were resuspended in EGM-2 Endothelial Cell Growth Medium-2 (Lonza, Alps, Swiss) and plated to the lower chamber to grow for 48 h at 37°C in order to form a monolayer. PDAC cells were resuspended in serum-free medium and applied to the upper chamber (2–6×10^4^ cells/well) without macrophages or with 6×10^4^ macrophages per well. EGM-2 containing 36 μg/mL of CSF1 (MedChemExpress, New Jersey, USA) was added to the bottom of chamber. The details referred to previously published literatures.^44^^,^^45^^,^^46^

### Quantification and statistical analysis

#### Statistical analysis

GraphPad Prism v8.0.1 and Statistical Package for Social Science (SPSS, v20.0) software were used for statistical analysis. Variables were expressed as mean ± SD. The results of two groups were compared using Student’s *t*-tests, while multiple comparisons were performed by one-way ANOVA. The survival curve of patients was calculated using the Kaplan–Meier method. *p*-value less than 0.05 was considered statistically significant. The Spearman’s correlation analysis was implemented to calculate the coefficients between the expression of TFAP2A and immune cell representative genes, and was visualized by Gephi software.

## Supplemental information

Document S1. Figures S1–S5

Table S1. FPKM values of protein coding genes in four stages of *in vitro* liver differentiation model, related to Figure 1

Table S2A. The RNA sequencing data of 11 pairs of PDAC tissues and their corresponding liver metastatic tissues, related to Figure 1 Table S2B. The log2(FKPM_1) values of specific genes, related to Figure 1 and S1.

Table S3. The gene list with similar expression patterns to HNF4A and FOXA1, related to Figure 1

Table S4. The enriched GO pathways of genes in Table S3, related to Figure 1

Table S5. Summary of clinical features of PDAC cases used for survival analysis, related to Figure 1

Table S6. The predicted binding sites of TFAP2A at the promoter region of MYC, related to Figure 4

Table S7. Sequence information of plasmids used in Dual-luciferase reporter assay, related to STAR Methods
