# Supplementary material for: TFAP2A enhances tumor stemness and promotes metastasis in pancreatic ductal adenocarcinoma
Source: iScience. 2025 Jul 5;28(8):113060. doi: 10.1016/j.isci.2025.113060 (PMC12302244; doi:10.1016/j.isci.2025.113060)
Supplement: Document S1. Figures S1–S5 [file mmc1.pdf]

iScience, Volume *28*

Supplemental information

TFAP2A enhances tumor stemness and promotes metastasis in pancreatic ductal adenocarcinoma

Jiaxin Luo, Zezhi Ding, Dongjie Chen, Yongsheng Jiang, Yizhi Cao, Minmin Shi, Xiaomei Tang, Jia Liu, Meilin Xue, Zehui Zhang, Kexian Li, Yu Bao, Fangfang Ma, Ting Wang, and Lingxi Jiang

Figure S1

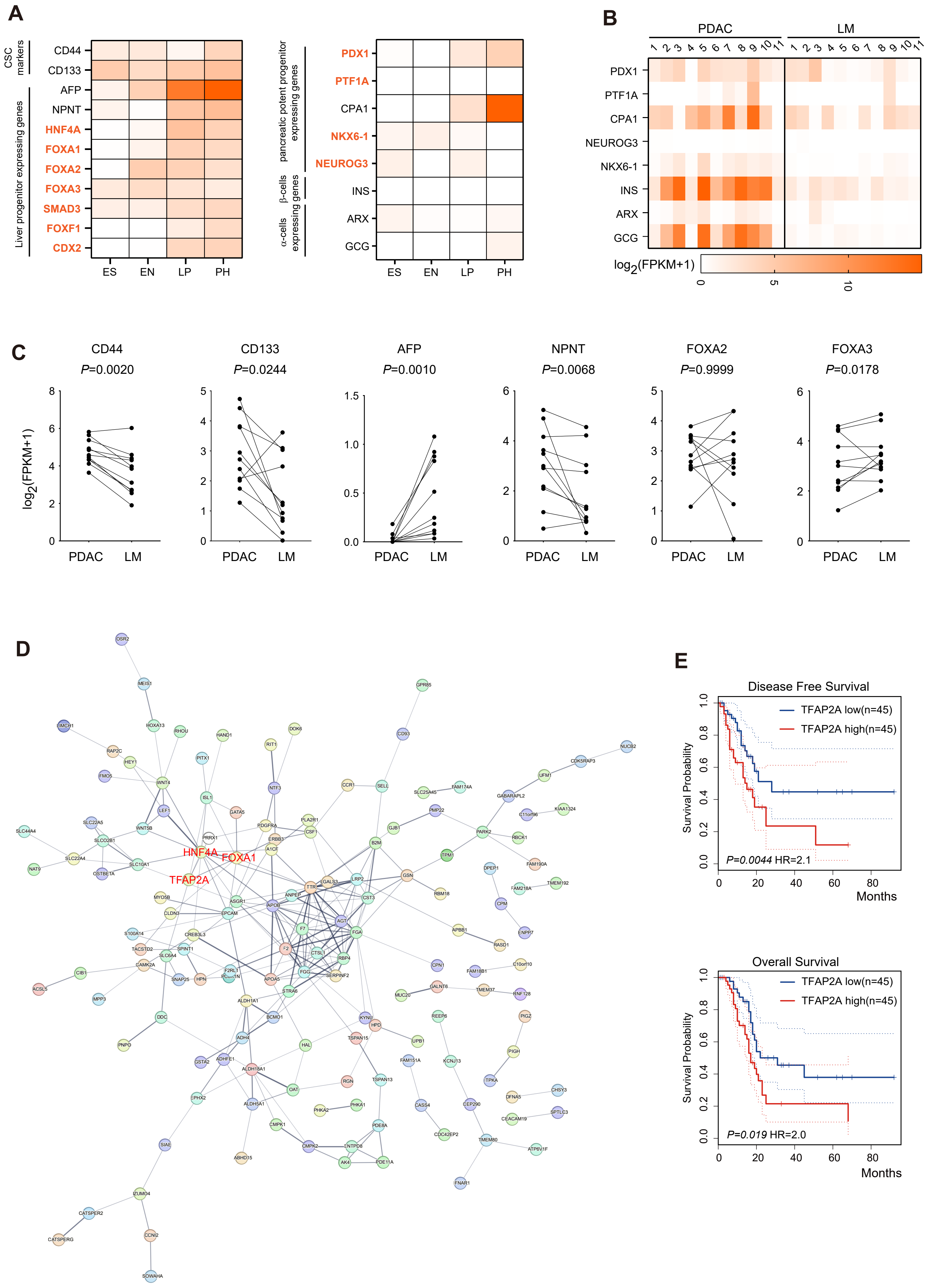
A
CSC markers
CD44
CD133
Liver progenitor expressing genes
AFP
NPNT
HNF4A
FOXA1
FOXA2
FOXA3
SMAD3
FOXF1
CDX2
ES EN LP PH
pancreatic potent progenitor expressing genes
PDX1
PTF1A
CPA1
NKX6-1
NEUROG3
β-cells genes
INS
α-cells expressing genes
ARX
GCG
B
PDAC
LM
1 2 3 4 5 6 7 8 9 10 11
PDX1
PTF1A
CPA1
NEUROG3
NKX6-1
INS
ARX
GCG
log2(FPKM+1)
0 5 10
C
CD44 P=0.0020
CD133 P=0.0244
AFP P=0.0010
NPNT P=0.0068
FOXA2 P=0.9999
FOXA3 P=0.0178
log2(FPKM+1)
PDAC LM
D
HNF4A
FOXA1
TFAP2A
E
Disease Free Survival
TFAP2A low(n=45)
TFAP2A high(n=45)
Survival Probability
P=0.0044 HR=2.1
Months
Overall Survival
TFAP2A low(n=45)
TFAP2A high(n=45)
P=0.019 HR=2.0
Months

Figure S1. Identification of highly expressed genes in liver progenitor of the in vitro liver differentiation model and clinical significance of TFAP2A expression, related to Figure 1. A. Heatmaps based on the expression levels of representative genes of CSC, LP, pancreatic potent progenitor, β cells and α cells in four stages from liver differentiation model. Related to Figures 1A. **B.** Heatmap of expression level of genes related to pancreas differentiation in primary PDAC and liver metastases tissues from 11 patients at Ruijin Hospital. **C.** Comparing the expression levels of CD44, CD133 and other highly expressed genes in LP between primary PDAC and liver metastases samples (n=11). **D.** The protein association network of selected genes which have similar expression pattern to HNF4A and FOXA2 at the four stages of in vitro liver differentiation model. **E.** Kaplan–Meier overall survival curve and disease-free survival curve of two PDAC groups in The Cancer Genome Atlas (TCGA). LM: liver metastasis.

Figure S2

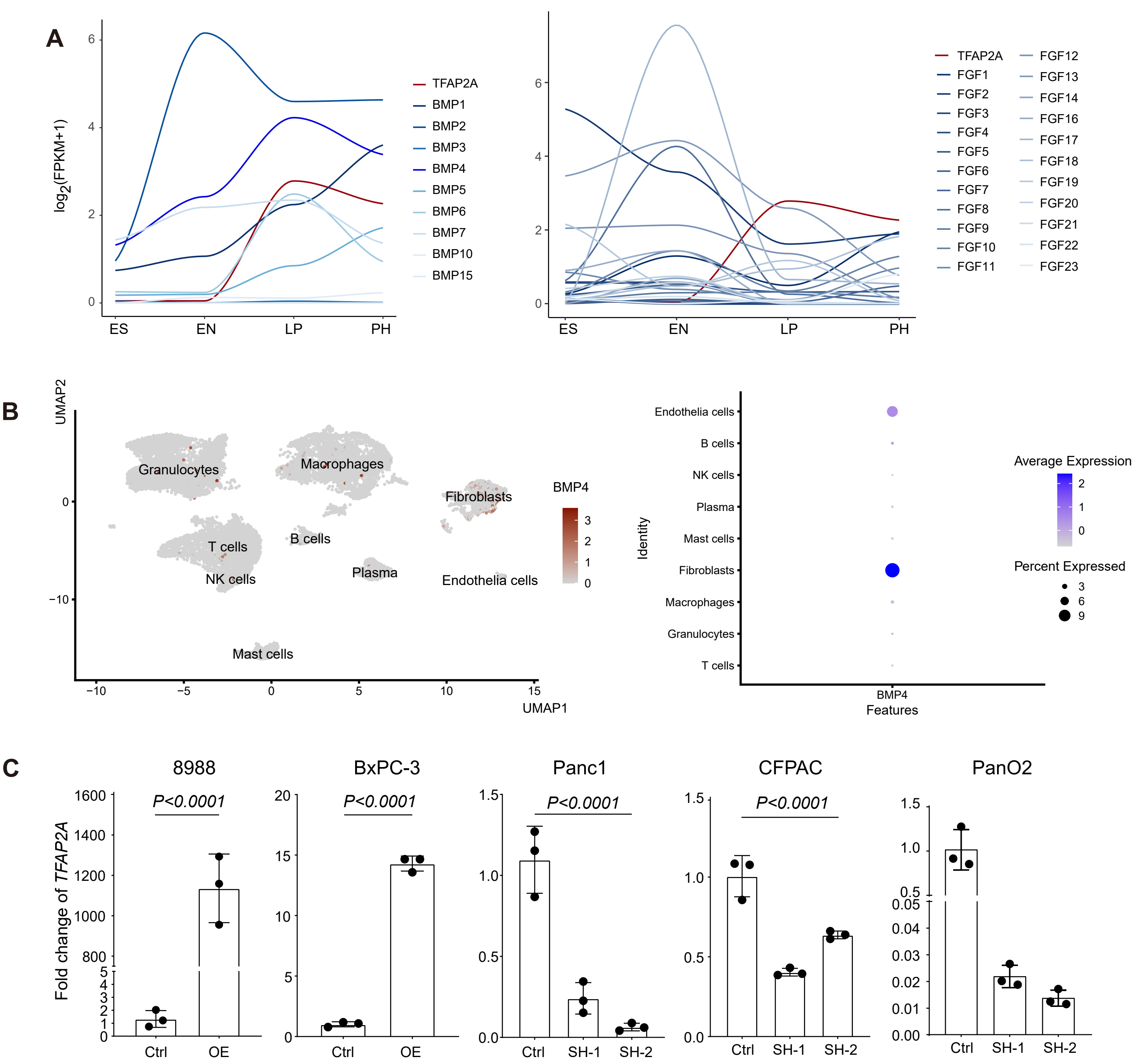

Figure S2. CAFs upregulate the expression of TFAP2A by BMP4, related to Figure 2. A. Expression levels of BMP and FGF family members in *in vitro* liver differentiation model. **B.** BMP4-associated single-cell analysis in the tumor microenvironment using data from the National Omics Data Encyclopedia (NODE, accession no. OEP003254). **C.** RT-qPCR was performed to determine the overexpression or knockdown of TFAP2A in 8988, BxPC-3, Panc1, CFPAC and PanO2 cells transfected with *TFAP2A* (OE), shRNA against *TFAP2A* (SH-1, SH-2), and corresponding control plasmids (Ctrl). *GAPDH* was used as control. Error bars represent the mean values ± standard deviation.

Figure S3

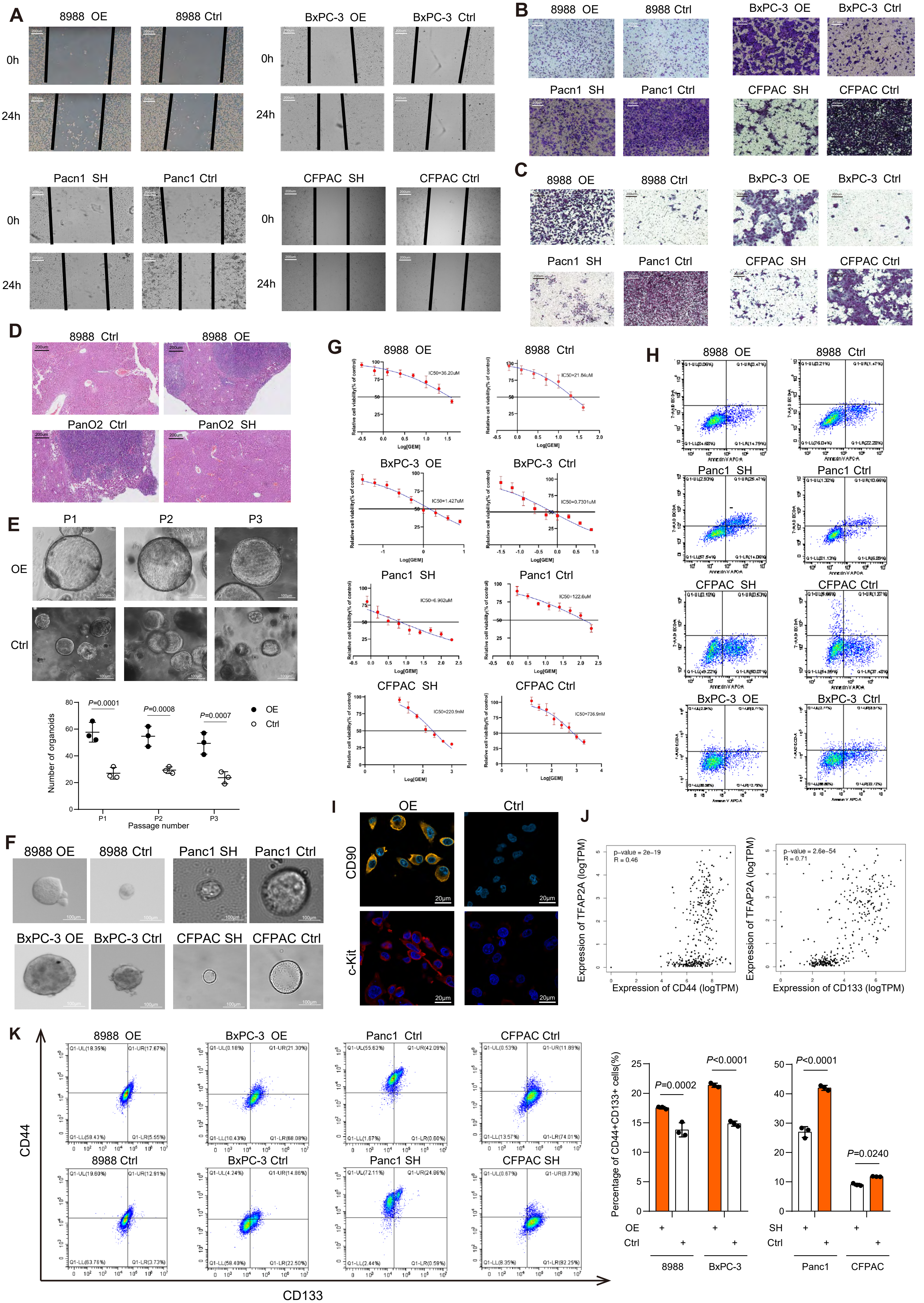
A
8988 OE
8988 Ctrl
BxPC-3 OE
BxPC-3 Ctrl
0h
24h
Pacn1 SH
Panc1 Ctrl
CFPAC SH
CFPAC Ctrl
B
C
D
8988 Ctrl
8988 OE
PanO2 Ctrl
PanO2 SH
E
P1
P2
P3
OE
Ctrl
P=0.0001
P=0.0008
P=0.0007
Number of organoids
Passage number
F
8988 OE
8988 Ctrl
Panc1 SH
Panc1 Ctrl
BxPC-3 OE
BxPC-3 Ctrl
CFPAC SH
CFPAC Ctrl
G
IC50=36.20uM
IC50=21.84uM
IC50=1.427uM
IC50=0.7301uM
IC50=6.962uM
IC50=122.6uM
IC50=220.9nM
IC50=736.9nM
Log[GEM]
Relative cell viability(% of control)
H
I
OE
Ctrl
CD90
c-Kit
20μm
J
p-value = 2e−19
R = 0.46
p-value = 2.6e−54
R = 0.71
Expression of TFAP2A (logTPM)
Expression of CD44 (logTPM)
Expression of CD133 (logTPM)
K
CD44
CD133
Percentage of CD44+CD133+ cells(%)
P=0.0002
P<0.0001
P<0.0001
P=0.0240
OE
Ctrl
SH
8988
BxPC-3
Panc1
CFPAC

Figure S3. TFAP2A enhances migration and stemness capabilities of PDAC cells, related to Figure 3. A-C. Representative images of wound healing assay (A), transwell assay (B) and invasion assay (C) (scale bar=200 μm). Related to Figures 3A-C. **D.** Representative H&E staining images of liver metastatic foci developed by TFAP2A overexpression, knockdown and control PDAC cells. OE: TFAP2A overexpression; SH: TFAP2A knockdown; Ctrl: control. Scale bar=200 μm. **E.** Representative images (top) and quantitative analysis (bottom) comparing the size and number of patient-derived organoids (passages 1-3) with or without TFAP2A overexpression. Scale bar=100 μm. **F.** Representative images of spheroids formed by TFAP2A overexpression or knockdown PDAC cell lines. Scale bar=100 μm. Related to Figure 3F. **G.** IC50 values of gemcitabine in 8988, BxPC-3, Panc1 and CFPAC cells. **H.** Representative images showing the percentage of apoptotic cells in 8988, BxPC-3, Panc1 and CFPAC cells. Cells were exposed to gemcitabine for 48 h at the IC50 concentration. Early apoptotic cells (Q1-LR) and late apoptotic cells (Q1-UR) were identified as the apoptotic cells. Related to Figure 3G. **I.** Representative images of the immunofluorescence staining of CD90 (yellow), c-kit (red) in PDAC cells. DAPI (blue) was used for nuclei counterstaining. Scale bar=20 μm. **J.** Correlation analysis of CD44 (left) or CD133 (right) and TFAP2A expression in PDAC from TCGA dataset. **K.** Flow cytometry plots (left) and summarized bar chart (right) of CD44 and CD133 in 8988, BxPC-3, Panc1 and CFPAC cell lines. OE: TFAP2A overexpression; SH: TFAP2A knockdown; Ctrl: control plasmids transfection. Three independent experiments were performed. Error bars represent the mean values ± standard deviation.

Figure S4

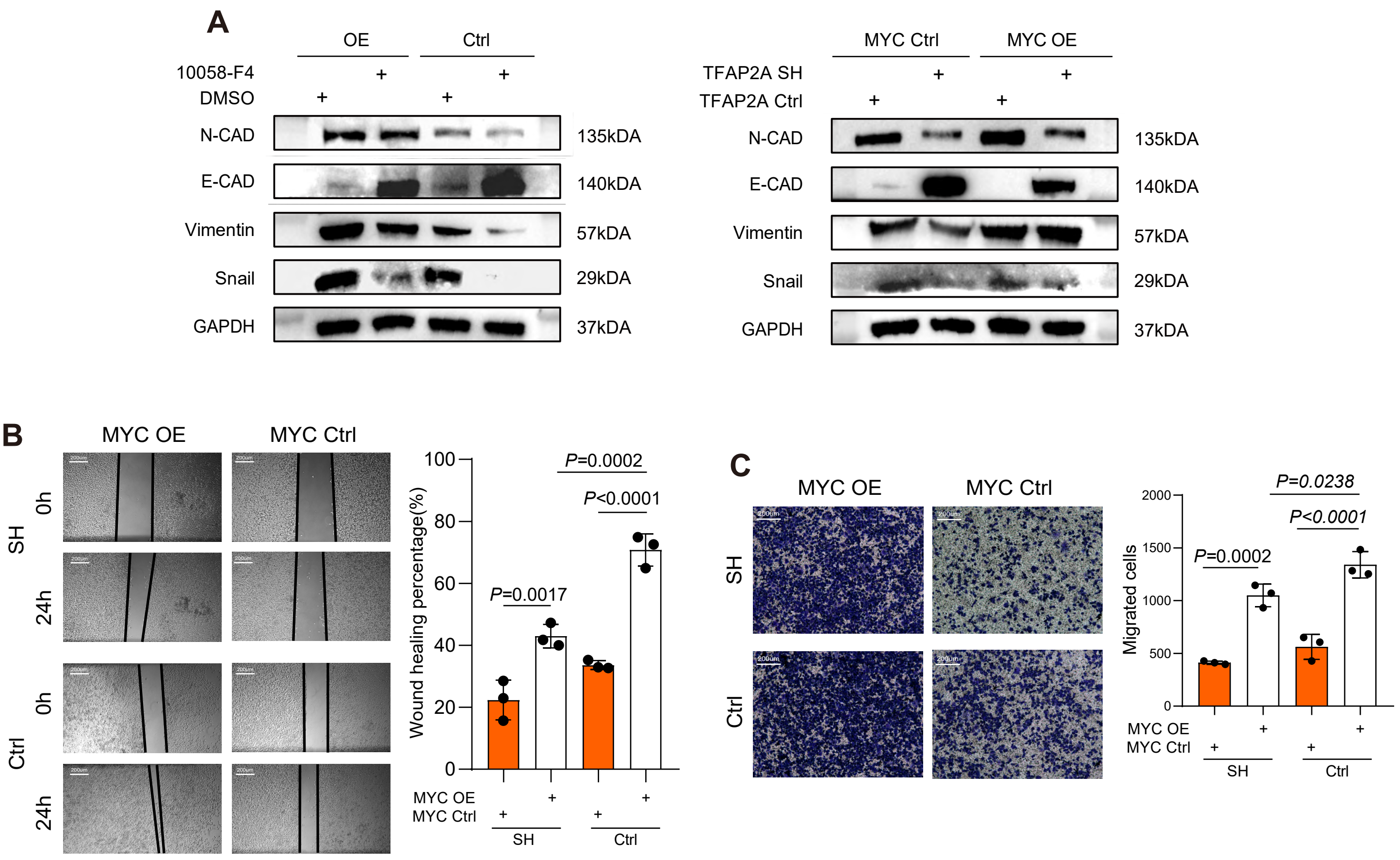

Figure S4. MYC enhances EMT process and migration capabilities in TFAP2A knockdown PDAC cells, related to Figure 4. A. The protein levels of EMT-related genes in 8988 cell line treated with or without 10058-F4 and Panc1 cell line transfected with MYC overexpression plasmid or control ones were determined by western blotting. GAPDH was used as loading control. Antibody dilution= 1:1000. **B, C.** Representative images (left) and summarized bar chart (right) of wound healing assay (**B**) and transwell assay (**C**) of Panc1 cell line transfected with MYC overexpression plasmid or control ones. Scale bar=200 μm. Three independent experiments were performed. Error bars represent the mean values ± SD.

Figure S5

A

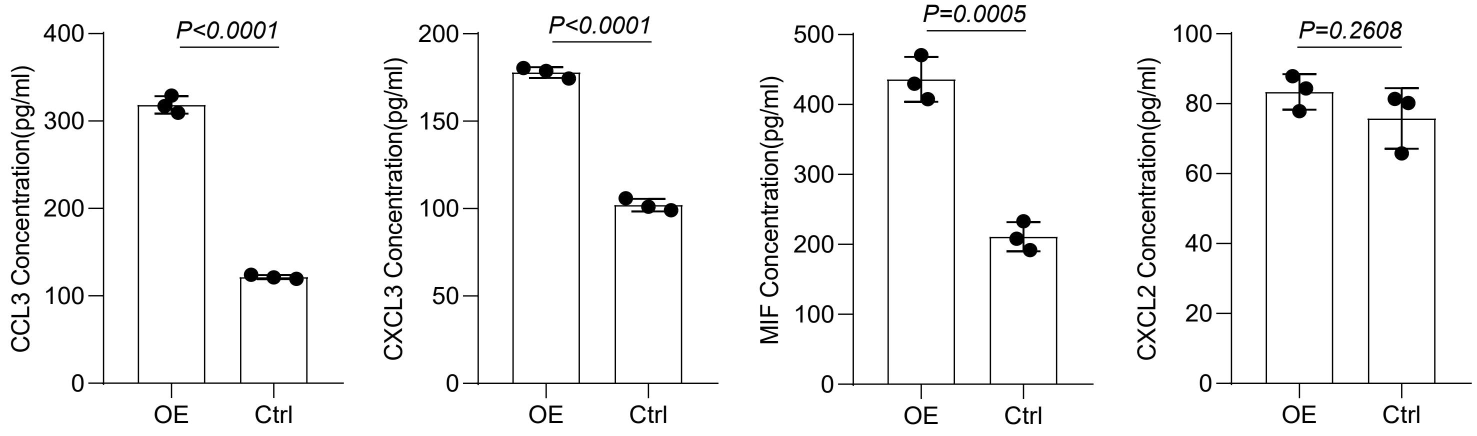

B

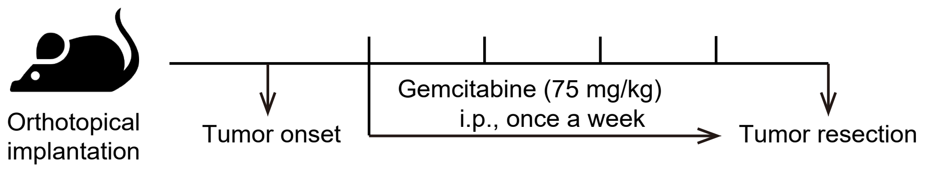

C

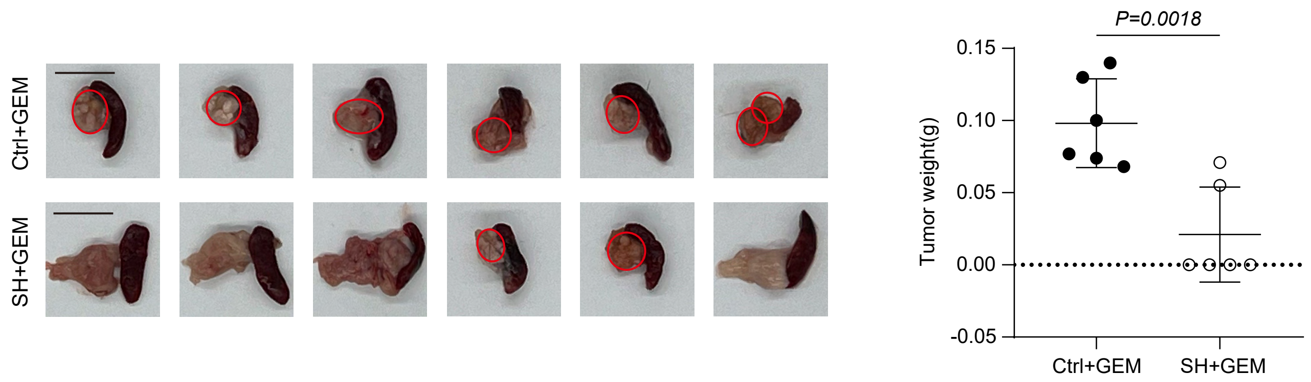

D

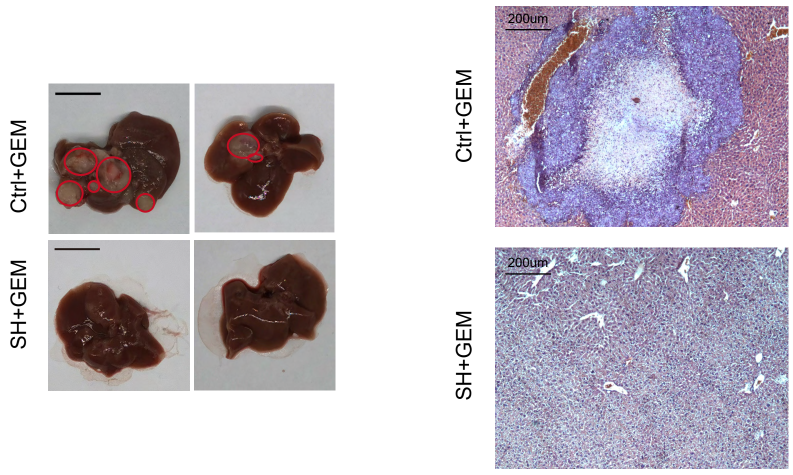

Figure S5. TFAP2A regulates chemokine secretion and pancreatic tumor gemcitabine response, related to Figure 5. A. The concentrations of CCL3, CXCL3, MIF, and CXCL2 in cellular supernatant from TFAP2A overexpression and control groups, detected by ELISA assays. OE: TFAP2A overexpression; Ctrl: control. Three independent experiments were performed. Error bars represent the mean values ± standard deviation. **B.** Schematic diagram and gemcitabine treatment strategy of orthotopic pancreatic xenograft from TFAP2A knockdown and control cells. **C.** Images of the tumor tissues (left) and quantitative analysis (right) showing the tumor weights of TFAP2A knockdown compared to control group, with gemcitabine treatment. n=6/group. SH: TFAP2A knockdown; Ctrl: control; GEM: gemcitabine. Scale bar=10mm. Error bars represent the mean values ± standard deviation. **D.** Representative images of gross (left) and H&E staining (right) of excised liver. Scale bar=200 μm.
